# Does an offer for a free on-line continuing medical education (CME) activity increase physician survey response rate? A randomized trial

**DOI:** 10.1186/1756-0500-5-129

**Published:** 2012-03-07

**Authors:** Anthony J Viera, Teresa Edwards

**Affiliations:** 1 Department of Family Medicine, University of North Carolina at Chapel Hill, Chapel Hill, NC, USA; 2 Odum Institute for Research in Social Science, University of North Carolina at Chapel Hill, Chapel Hill, NC, USA

## Abstract

**Background:**

Achieving a high response rate in a physician survey is challenging. Monetary incentives increase response rates but obviously add cost to a survey project. We wondered whether an offer of a free continuing medical education (CME) activity would be effective in improving survey response rate.

**Results:**

As part of a survey of a national sample of physicians, we randomized half to an offer for a free on-line CME activity upon completion of a web-based survey and the other half to no such offer. We compared response rates between the groups. A total of 1214 out of 8477 potentially eligible physicians responded to our survey, for an overall response rate of 14.3%. The response rate among the control group (no offer of CME credit) was 16.6%, while among those offered the CME opportunity, the response rate was 12.0% (*p *< 0.0001).

**Conclusions:**

An offer for a free on-line CME activity did not improve physician survey response rate. On the contrary, the offer for a free CME activity actually appeared to worsen the response rate.

## Background

Surveys of physicians are frequently used to assess attitudes, knowledge, and clinical practice related to health care and health services. As such, they are valuable tools to describe and understand variation in practice patterns. A high response rate is usually desired to ensure adequate generalizability. Unfortunately, achieving a high response rate in physician surveys often proves challenging. Research and anecdotal evidence indicate that motivating physicians to spend even a few minutes completing a survey is difficult [1-3].

Monetary incentives can help researchers improve response rates. A systematic review found that overall, monetary incentives doubled the odds that physicians would respond to a survey [4]. Some recent large surveys have given incentives of $50 [5,6]. Incentives alone can then add substantially (e.g., $97,400 in one recent survey [5]) to the cost of the study. It is not clear whether there is a certain threshold below which monetary incentives do not help to improve response rates. There appear to be little differences in response rates as the amount of monetary incentive increases between one dollar and $20 [4]. However, a randomized trial found a 16% higher response rate (68% vs 52%) among physicians mailed a $50 check compared to those mailed a $20 check [6]. Still, even a small monetary incentive can add significant costs to a survey project. Monetary lottery prize offerings are less costly, but also less effective [4].

A variety of non-monetary inducements have been tried, and have generally met with little success. Examples include pencils, stickers, candy, and computer programs [4]. Nonmonetary inducements are obviously attractive when considering budgetary limitations. Given the fact that physicians are required to maintain Continuing Medical Education (CME) credits, we wondered whether a CME incentive would help increase survey response rates among physicians. Little has been published on this potential strategy. We found only two papers, one in which CME credit plus a $5 incentive was reported to improve response rates, and another in which CME credits did not add effectiveness above and beyond a monetary incentive [4,7,8]. We decided to test whether the offer of a CME activity is effective at increasing response rates among a sample of United States (U.S.) physicians. We conducted an experiment to assess whether offering a free on-line CME activity without an individual monetary inducement would increase survey response rates. The survey was web-based, but the invitation to participate was sent by postal mail. In this paper, we describe the results of this experiment.

## Methods

### Population and sampling

Our target population was U.S. physicians from three specialties: family medicine, general internal medicine, and cardiology. The sampling frame was 9,000 physicians randomly drawn from the large mailing lists of the American Academy of Family Physicians (AAFP) and the American College of Physicians (ACP). A total of 3,000 physicians from each specialty were selected. We removed AAFP members who were subsequently identified as medical students.

### Survey design and implementation

The survey was web-based, contained a total of 40 items, and took approximately 10 minutes to complete. We chose to use a web-based survey rather than a paper survey for feasibility reasons. However, an earlier attempt to reach physicians via email proved unsuccessful because of extremely unreliable email addresses in a commercial database. We therefore decided to use a mixed methods approach combining mailed invitations with a web-based survey. The physicians were mailed a personalized letter describing the survey and its purpose to gather physicians' opinions on "new ideas in cardiovascular disease prevention." The main paper from the survey has been published elsewhere [9]. The letter contained a URL for accessing the survey via the internet. Each physician was assigned a personalized code to enable tracking of nonrespondents. The letters informed physicians they would receive a chance to be entered into a drawing for one of two $500 Visa gift cards if they completed the survey. We included this drawing to offer at least some small incentive to all the invited physicians, concerned that no incentive at all would lead to even lower response rates. Reminder letters were sent to nonrespondents at weeks 2 and 4.

### Experimental design

Prior to mailing the invitation letters, we randomly assigned each physician to a "CME offer" group or "no CME offer" group. Randomization was stratified by physician specialty. Thus, half of the sample members from each specialty were offered a chance to earn 2 hours of CME credit if they completed the survey. The letter to these physicians included the following paragraph:

"To thank you for your time, after you complete the questionnaire, you can participate in a free CME activity to earn up to 2 hours of Category 1 AMA Prescribed credit. At the conclusion of the questionnaire, we provide links to three articles we believe you will find very interesting and educational along with a 4-question multiple choice quiz that you can complete to receive CME credit. No research data is collected on your responses to the quiz. It was developed solely as a CME opportunity for you."

The CME activity did not have to be completed at the time of survey completion. The activity consisted of reading three articles that were relevant to the ideas in cardiovascular disease prevention that we were asking about in our survey. The articles were provided free of charge (permission-approved) via a web page. After reading the articles, participants completed a four question quiz. The activity was approved for up to 2 hours of Category 1 AMA Prescribed credit by the AAFP, but all physicians were eligible to receive such credit.

### Analysis

We calculated overall response rates and then compared response rates between the group offered the CME activity and the group not offered the CME activity. We also conducted analyses stratified by physician specialty and by geographic region (the only variables we had on nonrespondents). We tested for significant differences using chi-square.

### Study approval

This study was approved by the Office of Human Research Ethics Institutional Review Board of the University of North Carolina at Chapel Hill.

## Results

After removing those in the initial sample subsequently identified as medical students, 8607 physicians were invited to participate in the survey. Of the mailed invitation letters, 84 were returned as undeliverable, including 10 because the intended recipient was deceased. Three physicians called or emailed to decline participation (who we presumed eligible), and 46 contacted us to say they are retired and no longer see patients. A total of 1214 physicians participated in the survey. Thus, our total adjusted response rate was 14.3% (1214/8477).

Respondents were predominantly male (73%), spent more than 75% of work time in office based patient care (55%), and have been in practice for 10 years or more (79%) (Table 1). Small group practices were the most common practice setting (44%), and the most common region of the country practiced in was the South (34%).

**Table 1:** Characteristics of respondents*

	Percent
Specialty	
Family medicine	39
General internal medicine	27
Cardiology	29
Other	5
Male	73
Years in practice	
≥ 20	61
10-19	18
< 10	22
Region of country	
Northeast	24
South	34
Midwest	24
West	18
Time spent in office-based patient care	
≥75%	55
25 to 74%	34
< 25%	11
Practice setting	
Solo or small group	44
Large group	26
Academic/other	31

*N varies from 967 to 1000

Across all specialties, the response rate among the control group (no offer of CME credit) was 16.6%, while among those offered the CME opportunity, the response rate was actually *lower *at 12.0% (Table 2). Overall, family physicians were significantly more likely to respond than cardiologists or general internists (16.7% vs. 12.7% vs 13.9%, *p *< 0.0001). Within each specialty, the pattern of higher response among those who were not offered the quiz remained the same. The difference in response rates between groups was also consistent across all four geographic regions. Those offered the CME opportunity responded at consistently lower rates across the country.

**Table 2:** Response Rates (N = 8477)

	All	CME offer (n = 4245)	No CME offer (n = 4232)	*P*-value
		**%**	**%**	

Overall	14.3	12.0	16.6	< .0001

Specialty				
Family medicine	16.7	14.2	19.1	.0007
Internal medicine	13.9	12.4	15.5	.013
Cardiology	12.7	9.9	15.5	< .0001

Region of country				
Northeast	14.4	12.7	16.1	.03
South	14.1	12.0	16.1	.001
Midwest	15.2	12.7	17.6	.003
West	13.7	10.6	16.8	.0002

## Discussion

We sought to test whether an offer of a free web-based CME activity would increase response rates to a web-based survey. Somewhat surprisingly, if the CME offer had any effect, it appeared to be a deterrent to responding. While our response rate was low overall, it was even lower in the group who received the letter offering a free CME activity upon completion of the survey. The reasons for this are not clear. The overall low response rate suggests that physicians may not have been very interested in the topic. Although we indicated that the survey and the CME activity were not sponsored by any pharmaceutical company, it is possible that physicians still may have felt the activity was bound to be biased, perhaps designed to sway opinion. Another possibility is that physicians felt the CME activity would add to the time required to participate, although we tried to make it clear that the CME activity could be completed anytime within a year. Finally, the CME activity simply may have been viewed as something else that would have had to be done in their busy day, so it functioned as a disincentive.

One prior study that also surveyed U.S. family physicians, general internists, and cardiologists (as well as vascular surgeons) improved response rates after including an offer for CME credit after the second mail-out to nonrespondents [7]. However, that study also simultaneously included a $5 bill. It is therefore difficult to know whether it was the CME offer or the individual monetary incentive that improved initial nonrespondent response rates. Additionally, the survey was a mailed paper questionnaire as opposed to a web-based survey.

Another U.S. study included an offer of CME credit (7 to 10 credits) in exchange for completing a mailed paper questionnaire and reviewing some materials sent afterward [8]. That study also provided a $25 individual incentive to all respondents making it impossible to separate the effects of CME credit vs monetary incentive. The authors report that the CME credits had little overall effect based on comparison to response rates during pilot testing.

Our study adds to this limited body of literature because we compared a CME offer to no CME offer without individual monetary incentives. Our results should be interpreted in the context of some limitations, however. We tested a CME activity that used enduring materials (i.e., articles to read). Other forms of CME, such as web-based lecture, audio-cast, or similar downloadable material (e.g., podcast), might have different effects. Physicians may also be less enthusiastic about CME that is not interactive. Even with internet-based learning, interactivity and opportunities for repetition and practice improve learning outcomes [10], and our activity lacked these aspects. We also gave physicians no choice on the CME topic. An offer of choice of educational topic might have given potential respondents more confidence that the activity was not trying to "sell" them anything. Our use of a combined letter and web method meant physicians could not simply click on a link. This may have deterred some from responding, although should not have had differing effects across the two groups. Finally, our offer for the CME activity was in addition to the offer (which everyone received) of the chance to be entered into a gift card drawing. The effect of the CME offer alone (compared to no incentive at all) might have yielded different results, although this seems unlikely.

## Conclusions

In our study, an offer for a free on-line CME activity did not improve physician response rate in a design which used postal letters to invite participants to complete a web-based survey. On the contrary, the CME activity actually appeared to worsen response rate. The reasons for this are unclear. Qualitative research with physicians may be able to shed light on why a CME offer may or may not be effective.

## Competing interests

The authors declare that they have no competing interests.

## Authors' contributions

AJV conceived of the study and drafted the initial manuscript. TE oversaw the implementation of the study, prepared data files and analyses, and provided critical review and input into the manuscript. All authors read and approved the final manuscript.
